# Comparative Evaluation of Stroke Triage Algorithms for Emergency Medical Dispatchers (MeDS): Prospective Cohort Study Protocol

**DOI:** 10.1186/1471-2377-11-14

**Published:** 2011-01-27

**Authors:** Prasanthi Govindarajan, David Ghilarducci, Charles McCulloch, Jessica Pierog, Evan Bloom, Claiborne Johnston

**Affiliations:** 1 Department of Emergency Medicine, 505 Parnassus Avenue L 126 Mail Code 0208, University of California San Francisco, San Francisco, CA 94143-0208, USA; 2 American Medical Response EMS agency, 268 Calvin Place, Santa Cruz, CA 95060, USA; 3 Division of Biostatistics, UCSF Box 0560, 185 Berry Street, Lobby 5, Suite 5700 San Francisco, CA 94107-1762, USA; 4 Division of Emergency Medicine, 300 Pasteur Drive, Alway Bldg M121 Mail Code: 5119 Stanford, CA 94305-2200, USA; 5 Departments of Neurology, Epidemiology and Biostatistics, University of California San Francisco, USA

## Abstract

**Background:**

Stroke is a major cause of death and leading cause of disability in the United States. To maximize a stroke patient's chances of receiving thrombolytic treatment for acute ischemic stroke, it is important to improve prehospital recognition of stroke. However, it is known from published reports that emergency medical dispatchers (EMDs) using Card 28 of the Medical Priority Dispatch System protocols recognize stroke poorly. Therefore, to improve EMD's recognition of stroke, the National Association of Emergency Medical Dispatchers recently designed a new diagnostic stroke tool (Cincinnati Stroke Scale -CSS) to be used with Card 28. The objective of this study is to determine whether the addition of CSS improves diagnostic accuracy of stroke triage.

**Methods/Design:**

This prospective experimental study will be conducted during a one-year period in the 911 call center of Santa Clara County, CA. We will include callers aged ≥ 18 years with a chief complaint suggestive of stroke and second party callers (by-stander or family who are in close proximity to the patient and can administer the tool) ≥ 18 years of age. Life threatening calls will be excluded from the study. Card 28 questions will be administered to subjects who meet study criteria. After completion of Card 28, CSS tool will be administered to all calls. EMDs will record their initial assessment of a cerebro-vascular accident (stroke) after completion of Card 28 and their final assessment after completion of CSS. These assessments will be compared with the hospital discharge diagnosis (ICD-9 codes) recorded in the Office of Statewide Health Planning and Development (OSHPD) database after linking the EMD database and OSHPD database using probabilistic linkage. The primary analysis will compare the sensitivity of the two stroke protocols using logistic regression and generalizing estimating equations to account for clustering by EMDs. To detect a 15% difference in sensitivity between the two groups with 80% power, we will enroll a total of 370 subjects in this trial.

**Discussion:**

A three week pilot study was performed which demonstrated the feasibility of implementation of the study protocol.

## Background

Stroke is the third leading cause of death and a leading cause of long-term disability in the United States. Each year, approximately 800,000 suffer a new stroke, of which 87% are ischemic strokes. The estimated cost of stroke care in 2010 was $74 billion [1]. Thrombolysis with intravenous tissue plasminogen activator (IV t-PA) remains the only proven treatment for patients with acute ischemic stroke who present within 4 1/2 hours of symptom onset, in the absence of other contra-indications for treatment. However, the rate of thrombolysis for eligible patients nationally remains poor at about 4% [2]. While there are many factors that contribute to such a poor rate of IV t-PA use for acute ischemic stroke, we would like to focus on prehospital factors that aid in early stroke recognition and triage. Opportunities to recognize stroke in the prehospital setting occur during the first contact with 911 system i.e. interrogation by emergency medical dispatchers. Emergency Medical Dispatchers' role involves interrogating a 911 call, triaging based on caller complaints, recognizing the need for higher priority response and assigning the appropriate level of ambulance response. Based on available evidence, we know that emergency medical dispatcher using medical priority dispatch system Card 28 protocol have low rates of stroke recognition. Ellison and co-investigators reported a sensitivity of 61% and a specificity of 20% for stroke recognition by emergency medical dispatchers. Rosamond et al. reported that only 31% of patients discharged with a diagnosis of stroke/TIA were given a final assessment of stroke by emergency medical dispatchers using Card 28 protocol [3,4]. Further, it is known from published literature that the sensitivity of Card 28 for stroke recognition was approximately 40% [5]. Although, true sensitivity and specificity could not be assessed due to lack of system wide outcomes [3-6] based on available evidence, it is apparent that there is a need for a triage protocol for emergency medical dispatchers that could improve recognition of stroke.

Therefore, in order to increase recognition of true strokes by emergency medical dispatchers, the National Academy of Emergency Medical Dispatchers developed a stroke diagnostic tool to be used by the emergency medical dispatchers at the time of call interrogation. The stroke diagnostic tool uses questions from the Cincinnati Stroke Scale, which is an abbreviated National Institute of Health Stroke Scale (NIHSS) for use by paramedics. In the validation study performed by Kothari et al, Cincinnati Stroke Scale detected all anterior circulation strokes that were eligible for thrombolysis and had a sensitivity of 66% when performed by physicians on subjects with neurological symptoms [7,8]. Results of a more recent field validation study showed that Cincinnati Stroke Scale had a higher sensitivity than the original study. Bray et al reported a sensitivity of 95%, specificity of 56%, PPV of 85% and NPV of 79% [9].

Although infield validation of the Cincinnati Stroke Scale has been done, the performance of this when used by lay public with the assistance of emergency medical dispatcher' instructions over phone, has not been shown. Therefore, our study aims to

### Aim 1

To compare the *sensitivity *of Card 28 plus Cincinnati Stroke Scale versus Card 28 alone (defined as the proportion of subjects correctly diagnosed as stroke by emergency medical dispatcher to the total number of physician identified strokes, in ambulance transported subjects).

### Aim 2

To compare the *specificity *of Card 28 plus Cincinnati Stroke Scale versus Card 28 alone (defined as the proportion of subjects with no stroke to the total number of non-strokes assessments by physicians among ambulance transported patients).

This study will provide preliminary comparative data on the accuracy of the two triage protocols in detecting the number of patients triaged to primary stroke centers as well as those who receive IV t-PA at these centers.

## Methods/Design

### Overview of the study

The pilot study was approved and granted waiver of informed consent by the Committee of Human Research, University of California, San Francisco.

### Study design

This is a prospective cohort study to test the diagnostic accuracy of Card 28 alone versus Card 28 plus the Cincinnati Stroke Scale.

### Study setting

The study will be conducted over a one year period at the County Communication Center, Santa Clara, California. The County Communication Center was established in 1948 and serves the population of Campbell, Cupertino, Los Altos, Los Altos Hills, Los Gatos, Monte Sereno, Morgan Hill and Saratoga. The County Communication Center receives about 90,000 emergency medical dispatch calls every year. Most of these calls are 911 calls transferred from Public Service Answering Points (PSAPs) after determination of the need for interrogation of medical emergency. The study site is a National Academy of Emergency Medical Dispatchers (NAEMD) accredited center of excellence since 2002 [10].

### Study population

The target study population consists of all 911 callers with symptoms suggestive of stroke in the participating county. Specifically, the accessible study population includes subjects within the participating county with symptoms suggestive of stroke whose 911 calls are answered and interrogated by the emergency medical dispatchers at the County Communication Center, Santa Clara.

### Inclusion criteria

(1) All 911 calls transferred by local Public Service Answering Points (PSAPs) to the County Communication Center of Santa Clara County where the emergency medical dispatchers complete the interrogation using Medical Priority Dispatch System (MPDS) protocols.

(2) All 911 calls received directly at the County Communication center of Santa Clara County where emergency medical dispatchers complete the interrogation using the MPDS protocols.

(3) All 911 calls received from subjects (patient) aged ≥ 18 years or second party calls (by-stander or family who are in close proximity to the patient and can administer the tool) by subjects ≥ 18 years of age.

### Exclusion criteria

(1) All calls that require immediate response (ECHO level determinant for life threatening conditions such as unconsciousness, breathing difficulty) and emergency medical dispatchers cannot complete Card 28

(2) Calls answered by emergency medical dispatchers who have not completed training on the use of Cincinnati Stroke Scale.

(3) Calls originating from the cities of Palo Alto, Mountain View, Sunnyvale, Santa Clara, and San Jose that are not interrogated by the County Communication Center for Santa Clara County.

### Subject recruitment and enrollment

Patient eligibility will be determined by the emergency medical dispatchers at the time of the 911 call. No informed consent will be obtained due to the emergency nature of the call. The study protocol was reviewed and approved by the Institutional Review Board and was granted waiver of informed consent for the study.

### Study procedures

**Figure 1:**
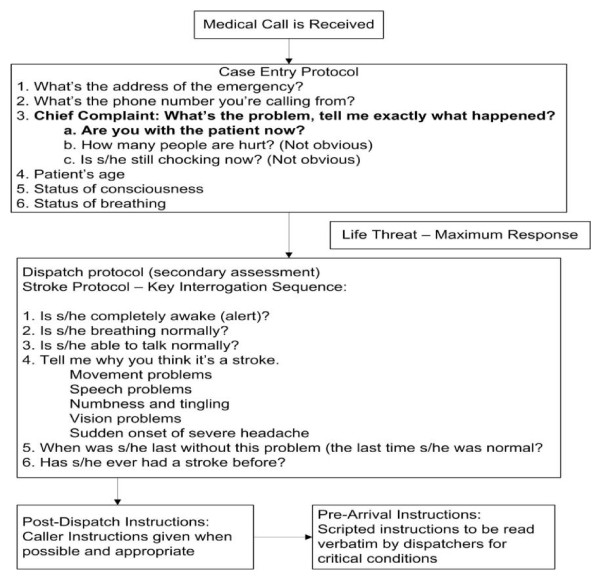
**Case entry protocol**. If caller reports any of the symptoms listed in question 4 (after reporting a chief compliant suggestive of stroke), dispatch determinant of stroke will be assigned. If none of the symptoms are reported (and chief compliant not suggestive of stroke, non-stroke dispatch determinant will be assigned).

Prior to initiation of the study, all emergency medical dispatchers in the County Communication Center will undergo training in the use of the Cincinnati Stroke Scale as well as in interpreting the findings relayed by the caller. During the study period, emergency medical dispatchers will continue to use the MPDS protocols for triaging a 911 call and assigning an ambulance. For calls that are suggestive of stroke, they will complete the Card 28 questions (Figure 1: **Card 28 Protocol For Emergency Medical Dispatchers**) followed by the scripted version of the Cincinnati Stroke Scale

**Figure 2:**
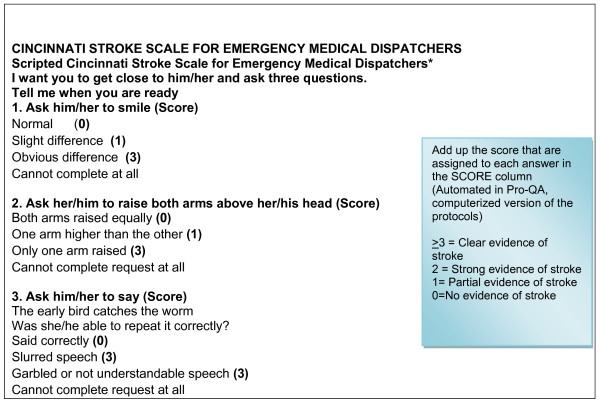
**Cincinnati stroke scale for emergency medical dispatchers**.

**(**Figure 2** - Cincinnati stroke scale for emergency medical dispatchers)**.

Emergency medical dispatchers will determine eligibility of the call based on chief compliant of symptoms suggestive of stroke. Common expressions used by callers to report symptoms of stroke include altered mental status, "stroke", trouble walking, impaired speech, falling or dizziness, muscle weakness and/or facial numbness [3]. If the emergency medical dispatcher determines the chief compliant to be a stroke related symptom, they will also screen for other life threatening symptoms. If associated life threatening symptom such as trouble breathing is reported, an ambulance will be dispatched immediately. These calls will be excluded since Card 28 will not be used to interrogate these 911 calls. We will also exclude calls which are placed by callers not in close proximity to the subject (i.e. third party caller like a family member calling from an office to report symptoms of their parent situated in their residence) and cannot administer the Cincinnati Stroke Scale. Since non-stroke causes of the symptoms (shown above) are more common in children and also because the ability of children to comprehend and administer the Cincinnati Stroke Scale to a subject is unknown, we will exclude subjects and callers less than 18 years of age from this study.

Once the emergency medical dispatcher determines that the call meets the inclusion criteria, they will interrogate the caller using the questions in Card 28. After completion of question 4 in the key interrogation sequence in Card 28 and recording of the initial diagnostic determinant of stroke (Figure 1), the scripted Cincinnati Stroke Scale tool will appear for use by the emergency medical dispatchers, in the Pro-QA system (computerized version of MPDS protocol). An assessment will be recorded electronically by the emergency medical dispatchers after completion of Cincinnati Stroke Scale. If during the interrogation of the call, emergency medical dispatchers determine that symptoms are not suggestive of stroke, they will complete the call by using one of the other medical priority dispatch protocol cards. Cincinnati Stroke Scale will also be applied to these calls and assessments recorded at the end of interrogation.

**Figure 3:**
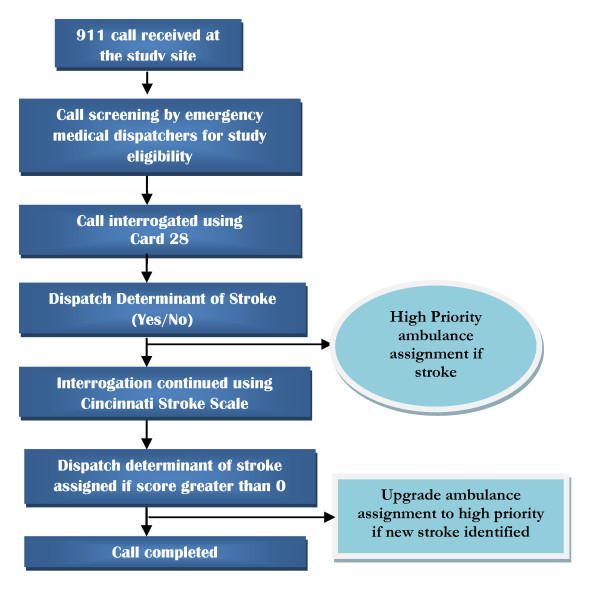
**Schematic representation of the study protocol**.

**(**Figure 3**: Schematic representation of the study protocol)**

This information will be used to create a screening log, which will be compared to all dispatch calls with chief complaints related to stroke symptoms to ensure eligible patients were not missed by the emergency medical dispatchers.

### Study measurements

During the study period, we will record the following prehospital variables. The source of the data variables is the computer assisted dispatch (CAD) database, which will capture all the listed variables during the 911 call. Variables will include the demographics of the subject (age, sex, location of the caller) the chief complaint of the caller; use of Card 28 based on the chief complaint of the caller; assessments after Card 28 which will be listed as dispatch determinant of stroke (CVA-28), assessment following completion of Cincinnati Stroke Scale, time from initiation of Card 28 to completion of key questions in the protocol and time from initiation to completion of Cincinnati Stroke Scale.

### Study outcomes

The primary study outcome is the hospital based diagnosis of all subjects who were interrogated with Card 28 and Cincinnati Stroke Scale by emergency medical dispatchers. The secondary study outcomes include time to complete the two stroke protocols and rate of intravenous t-PA use in this cohort.

### Data quality and management

The prehospital agency and OSHPD data will be encrypted and shipped to the Database Management Unit of Academic Research Systems, a unit of the Clinical and Translational Science Institute, University of California San Francisco. The data will be converted to SAS tables and visually inspected for inconsistencies. Using a subset of the data, probabilistic linkage will be used to link the databases (Figure 3) [11,12]. The merged dataset will be de-identified and will be used for statistical analysis.

### Linking databases

**Figure 4:**
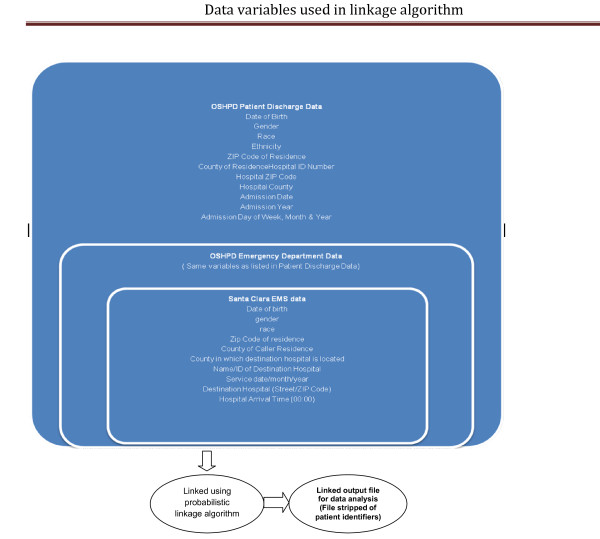
**Data variables used in probabilistic matching algorithm**.

**(**Figure 4**: Data variables used in probabilistic matching algorithm)**

The primary source of individual patient outcome data is the discharge abstract file of OSHPD that also contains variables like age, date of birth, sex, race/ethnicity, zip code, county of residence, hospital zip code, admission date, month and year, principal emergency department and discharge diagnosis, principal emergency department and hospital procedure codes, and discharge date. Prehospital database contains the following data variables: date of birth, gender, race, zip Code of residence, hospital ID, date of service, county of caller residence, zip code of caller residence, county in which destination hospital is located and the service date/month/year. These variables will be used to link the prehospital data with the outcome database. The details of the methodology will be published in another paper but preliminary results of the linkage algorithm showed close to 90% unique matches.

### Data analysis plan

The primary analysis will compare the sensitivity and specificity of the two protocols using logistic regression and generalized estimating equations to adjust for clustering by dispatcher. Wald test will be used to test the significance of the coefficients in the regression model. The data variables (listed in procedures section) in the computer assisted dispatch database and OSHPD database will be used to link the two databases and to calculate the diagnostic accuracy of the triage protocols. The hospital discharge diagnosis will be used as the gold standard for calculation of the performance characteristics of the protocols. Use of IV t-PA in the true stroke population will be determined by the procedure code in OSHPD and will be compared between the two protocol groups.

For the primary analysis, sensitivity will be defined as the proportion of true positives transported by EMS (stroke diagnosis by both emergency medical dispatchers and hospital discharge diagnosis) to the total number of patients with a hospital based stroke diagnosis. Specificity will be defined as the proportion of true negatives (no diagnosis of stroke by emergency medical dispatchers providers and non-stroke discharge diagnosis) to the total number of patients with hospital based non stroke diagnosis given by physicians (table 1).

**Table 1:** Variables used in measurement of test characteristics of Card 28 alone and Card 28 and Cincinnati Stroke Scale

Dispatcher Recognition Of Stroke - Card 28	Hospital Discharge Diagnosis of Stroke	
	**Positive**	**Negative**

**Positive**	*TP*	*FP*

**Negative**	*FN*	*TN*

Dispatcher Recognition Of Stroke - Card 28 and Cincinnati Stroke Scale	Hospital Discharge Diagnosis of Stroke	

	**Positive**	**Negative**

**Positive**	*TP*	*FP*

**Negative**	*FN*	*TN*

### Sample size calculations

During the one year study period, we anticipate about 90,000 emergency medical dispatch calls and about 350 eligible stroke calls. Based on published data showing a sensitivity of 40% for emergency medical dispatchers using MPDS protocol, we will have 80% power to detect an absolute 15% greater positive predictive value for emergency medical dispatchers stroke recognition using Cincinnati Stroke Scale with a two-sided alpha = 0.05

## Discussion

Rigorous outcome based assessments of new protocols is a rare occurrence in prehospital medicine. In our study, we will determine the test characteristics of the protocols and the time taken to administer them using hospital based outcomes obtained from state administrative database and linked to prehospital database using probabilistic linkage methodology. Our approach is innovative because this is a system-wide study to determine the test characteristics of the protocols. In addition to providing evidence on the performance characteristics of the triage protocols, the study will provide preliminary data on the rate of use of IV t-PA among groups triaged using the two different protocols and thereby provide some insight into early recognition of stroke and clinical outcomes for stroke patients.

Apart from the strengths discussed above, we recognize some of the limitations in the study design. One of the limitations includes lack of generalizability to all dispatch centers using medical priority dispatch system protocols. The County Communication is a center of excellence with high compliance to the protocols and is one among the 103 accredited centers worldwide. Therefore, the training, compliance to completion of protocols and data capture may be better than most dispatch centers thereby showing better results with the Cincinnati Stroke Scale.

The second limitation is that, we will not conduct a head-to-head comparison between Card 28 and the Cincinnati Stroke Scale to assess the diagnostic accuracies of these tools. Since the standard of care now is to use Card 28 algorithm for stroke, we cannot compare it to Cincinnati Stroke Scale alone. However, if our study results show that Cincinnati Stroke Scale closely matches the diagnostic accuracy of the Card 28, to the next step would involve studying the rate of use of intravenous t-PA among these groups.

Thirdly, since we will be using probabilistic linkage to link the databases, we will have outcome data that do not have an exact match in the prehospital dataset. The limitation of this method is that it may not provide a 100% match but has shown to have high sensitivity and specificity in studies involving prehospital databases with other registries [12]. On the other hand, the alternate approach, which involves following all callers/subjects to their destination hospitals in the County of Santa Clara to obtain their discharge diagnosis, may be impractical and due to privacy laws that limit sharing of information between hospitals and prehospital agencies. Further, this alternate technique is beyond the scope of this prehospital study and may not offer any more advantage compared to the results of the linkage process.

Lastly, while we will collect preliminary data on outcomes, our study is designed to assess the diagnostic accuracy of the two triage protocols and not powered to assess clinical outcomes between the two protocol groups.

## Conclusions

In this article, we have presented a prospective prehospital study protocol designed to compare the diagnostic accuracy of two stroke triage protocols used by emergency medical dispatcher. The results of the study are likely to show if Card 28 alone performs better than the combination of Card 28 and Cincinnati Stroke Scale as well as provide preliminary data on clinical outcomes for subjects triaged using a combination of protocols. We strongly believe that outcomes based assessment of new protocols will lead to creation of a model that combines the practice of evidence based development and implementation of triage protocols in the prehospital setting.

## Abbreviations

IV t-PA: Intravenous tissue plasminogen activator; NIHSS: National Institutes of Health Stroke Scale; CAD: Computer Assisted Dispatch Database; OSHPD: Office of Statewide Health Planning and Development; EMS: Emergency Medical Services; PSAP: Public Service Answering Point; CVA: Cerebrovascular accident; MPDS: Medical Priority Dispatch Systems;

## Competing interests

The authors declare that they have no competing interests.

## Authors' contributions

PG conceived and participated in the design and co-ordination of the pilot trial. SCJ and DG made substantial contribution to the design of the study. CM contributed to the study design and analysis. JP and EB contributed to acquisition and analysis of the pilot data. PG drafted the manuscript, all authors contributed to the manuscript revision and read and approved the final manuscript.

## Pre-publication history

The pre-publication history for this paper can be accessed here:

http://www.biomedcentral.com/1471-2377/11/14/prepub
